# Can education improve clinical practice concerning delirium in older hospitalised patients? Results of a pre-test post-test study on an educational intervention for nursing staff

**DOI:** 10.1186/s12909-018-1177-3

**Published:** 2018-04-02

**Authors:** Eveline L. van Velthuijsen, Sandra M. G. Zwakhalen, Ron M. J. Warnier, Ton Ambergen, Wubbo J. Mulder, Frans R. J. Verhey, Gertrudis I. J. M. Kempen

**Affiliations:** 1 Care and Public Health Research Institute (CAPHRI) and Department of Health Services Research, Maastricht University, P.O. Box 616, 6200 MD Maastricht, The Netherlands; 2 Department of Internal Medicine, Maastricht University Medical Centre, P.O. Box 5800, 6202 HX, Maastricht, The Netherlands; 3 Department of Methodology and Statistics, Maastricht University, Maastricht, The Netherlands; 4 Alzheimer Centre Limburg, MHeNS School for Mental Health and NeuroScience and Department of Psychiatry and Neuropsychology Maastricht University, P.O. Box 616, 6200 MD Maastricht, The Netherlands

**Keywords:** Delirium, Educational intervention, PRECEDE model, Hospital

## Abstract

**Background:**

Delirium is a common and serious complication of hospitalisation in older adults. It can lead to prolonged hospital stay, institutionalisation, and even death. However, it often remains unrecognised or is not managed adequately. The aim of this study was to evaluate the effects of an educational intervention for nursing staff on three aspects of clinical practice concerning delirium in older hospitalised patients: the frequency and correctness of screening for delirium using the 13-item Delirium Observation Screening score (DOS), and the frequency of geriatric consultations requested for older patients. The a priori expectations were that there would be an increase in all three of these outcomes.

**Methods:**

We designed an educational intervention and implemented this on two inpatient hospital units. Before providing the educational session, the nursing staff was asked to fill out two questionnaires about delirium in older hospitalised patients. The educational session was then tailored to each unit based on the results of these questionnaires. Additionally, posters and flyers with information on the screening and management of delirium were provided and participants were shown where to find additional information. Relevant data (outcomes, demographics and background patient data) were collected retrospectively from digital medical files. Data was retrospectively collected for four different time points: three pre-test and one post-test.

**Results:**

There was a significant increase in frequency of delirium screening (*P* = 0.001), and both units showed an increase in the correctness of the screening. No significant effect of the educational intervention was found for the proportion of patients who received a geriatric consultation (*P* = 0.083).

**Conclusion:**

The educational intervention was fairly successful in making positive changes in clinical practice: after the educational session an improvement in the frequency and correctness of screening for delirium was observed. A trend, though not significant, towards an increase in the proportion of geriatric consultations for older hospitalised patients was also observed.

## Background

Delirium was first described by Hippocrates about 2.500 years ago. However, despite its long history, the disorder still remains greatly unrecognised in older hospitalised patients, and management is often not done in accordance to the national or international guidelines [1, 2]. This failure to recognise or adequately manage a delirium is partially attributable to a lack of the necessary knowledge and skills among the medical and nursing staff [3–5]. Indeed, medical and nursing schools often do not put sufficient emphasis on the education of delirium in their curriculum [6].

Delirium is an acute brain failure and is a serious and common disorder in, older (65+) hospitalised patients. The Diagnostic and Statistical Manual of Mental Disorders 4^th^ edition (DSM-IV) [7] defines delirium as an acute (i.e. within hours or days) change in consciousness (i.e. a decreased sense of awareness of the surroundings) accompanied by a reduced ability to focus, sustain or shift attention, and a decline in cognitive functions (such as memory loss, disorientation or language disorders). The disorder is also often accompanied by perceptual disturbances, such as audio-visual hallucinations and delusions, and motor symptoms, such as agitation or slowing of movement and apathy [7]. It is associated with increased mortality, longer hospital stay, slower functional recovery, more re-admissions, and increased chances of developing dementia [1]. Delirium affects between 29 and 64% of older patients in hospital [1], yet only 2,5% to 5% are adequately diagnosed or reported [2, 8–10]. Timely recognition and adequate management should therefore be an important part of hospital care, especially for older hospital patients at risk of developing a delirium. The nursing staff is especially important in this process of recognition and management: they spend more time with patients than physicians do, and are often more likely to see sudden changes in behaviour or cognition. Some barriers for this failure to detect or adequately manage delirium in older hospitalised patients are a lack of knowledge and a negative attitude towards delirium [11]. Educational interventions could possibly have, therefore, the best results when aimed at the nursing staff.

Educational interventions aimed at increasing delirium knowledge in nursing staff have been previously developed and studied [12], including e-learning [13, 14], where the main outcomes were mostly related to an increase in knowledge measured with a questionnaire [12–14]. However, if one wants to bring about a change in clinical practice, it may be not enough to just increase knowledge on a certain topic [12]. According to the PRECEDE model (Predisposing, Reinforcing and Enabling Constructs in Educational Diagnosis and Evaluation) developed by Green and colleagues, an educational intervention may only become effective (i.e. bring about a change in clinical practice) when it should not only provide information, but also should reinforce and enable participants to use the acquired knowledge (i.e. by using reminders and feedback, providing flowcharts, guidelines etc.) [15].

The goal for the present study was to evaluate the effects of an educational intervention (based on the PRECEDE model) for nursing staff on the proportion of patients screened for delirium, the appropriate use of this screening and the proportion of patients being referred for a geriatric consultation. The a priori expectations were that there will be an increase in proportion of patients screened for delirium, an increase in the correctness of the screening, and an increase in geriatric consultations.

## Methods

### Design and setting

A pre-test post-test study was carried out at two inpatient medical units of the Maastricht University Medical Centre+ (MUMC+), a 715 bed university teaching hospital in the south of the Netherlands.

### Participants

The nursing staff from two inpatient units participated in the educational intervention: a combined unit (including dermatology, plastic surgery, nuclear medicine and urology, henceforth referred to as unit A) and a cardiology unit (henceforth referred to as unit B). Twenty six participants worked at unit A, and 33 participants at unit B. No inclusion criteria were set for the nursing staff to participate.

### Educational intervention

**Fig. 1:**
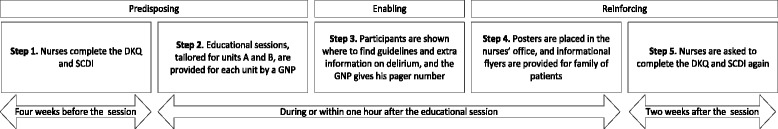
Workflow of the educational intervention. The top layer shows the different components of the PRECEDE model. The middle layer shows the five individual steps in the intervention. The bottom layer shows the time span of the educational intervention DKQ = Delirium Knowledge questionnaire, SCDI = Strain of Care for Delirium Index, GNP = Geriatric Nurse Practitioner

The main targets of the educational intervention were adequate recognition of delirium and adequate management of delirium in older hospitalised patients, in accordance with the Dutch guideline for delirium [16]. To achieve these targets, the educational intervention provided information on iatrogenic risk factors of delirium and how these could be avoided, what the guidelines state on the importance of screening for delirium, the symptoms of delirium, and delirium management. The educational intervention consisted of five different steps, in which the three components of the PRECEDE model (predisposing, reinforcing, and enabling) are incorporated. Figure 1 provides an overview of the educational intervention, including the different components of the PRECEDE model and a timeline of the individual steps. In step 1, the nursing staff of the two units were invited to complete two questionnaires: the Delirium Knowledge Questionnaire (DKQ) and the Strain in Care for Delirium Index (SCDI). The DKQ was designed by Detroyer and colleagues [14] and was used to assess existing levels of knowledge on delirium of the nursing staff. The SCDI was developed by Milisen and colleagues [17], and was used to assess the perceived burden for caring for delirious patients. In step 2, the educational session was given. The educational session was tailored to each unit according to the knowledge gaps made apparent by the questionnaires from step 1. The session included information on risk factors, precipitating factors, preventive measures, and delirium management. It was presented using slides, videos of patients with a hyperactive or hypoactive delirium, examples from clinical practice, and an interactive discussion. In step 3, which occurred at the end of the educational session, the Geriatric Nurse Practitioner (GNP), specialised in delirium, providing the educational session (author RMJW) showed the participants how and where to find the guidelines and more information about delirium on the hospital’s intranet. The GNP also gave his contact number so the nursing staff could always contact him concerning questions or doubts related to delirium in older patients. In step 4, directly after the educational session, posters about screening and management of delirium were placed in the nurses’ office. Additionally, informational flyers for informal caregivers were placed on the units (all educational material (slides, posters and flyers) is in Dutch, and is available upon request from the corresponding authors). In step 5, two weeks after attending the educational session, the nursing staff was asked to fill out the DKQ and SCDI again.

### Providing the educational sessions

Before planning the educational sessions, authors ELvV and RMJW consulted the individual unit leaders about when and where the educational session could best be held to maximise the reach of the educational session. For unit A, the unit leader decided it was best to incorporate the educational session in a monthly mandatory unit meeting. For unit B, it was decided to hold the session twice on two different dates, and to make participation voluntary. Thus, one session was held for unit A and two sessions were held for unit B. All participants attended the session only once. The educational sessions were delivered in March and April 2017.

### Data collection

#### Procedure

All data was collected by author ELvV. The data collected from patient files (i.e. three outcomes and patient demographics) was collected retrospectively in June 2017.

Data was collected for four different time points: three pre-test (February 2016, May 2016 and February 2017) and one post-test (May 2017). We included three pre-test measurements spread across a longer time period to be able to identify any pre-existing trends or seasonal differences in the outcome measures. Patient files were included for data collection if the patients were 70 years or older at time of hospital admission and were admitted to either unit A or unit B.

#### Main outcomes

The main aim of the educational intervention was to improve the care for delirious patients in the MUMC+ by improving adherence to the Dutch guideline for the care of delirious hospitalised patients. These improvements focussed mainly on screening for delirium and on how to manage a delirious patient. As the guidelines are aimed at both the medical and nursing staff, we selected those parts of the guideline which were applicable to the nursing staff and could be obtained through the patient’s digital medical records retrospectively. The main outcomes of the study were: 1. the proportion of older patients on the unit screened for delirium using the 13-item Delirium Observation Screening score (DOS); 2. the number of patients for whom the DOS was completed in accordance with the guideline and the original developers [18] (i.e. three times a day for at least three consecutive days (henceforth referred to as DOS “performed correctly”)); and 3. the proportion of older patients receiving a geriatric consultation.

All DOS scores and the date and time at which the DOS was filled out are all recorded in the patients’ medical file. The DOS score is initiated either by the nursing staff, a physician or by a GNP after a geriatric consultation. The criteria for keeping DOS scores is when patients are delirious or at risk of developing a delirium. A DOS score was considered to be performed correctly if it was completed three times a day for at least three consecutive days [18]. The third outcome was the proportion of patients receiving a geriatric consultation. Nursing staff can request a geriatric consultation with a GNP whenever they feel this is necessary. During a geriatric consultation, a GNP visits the patient for an assessment of frailty and risk of delirium. After the consultation, advice on all factors concerning geriatric care (hydration, nutrition, physical therapy, fall prevention etc.) is given to the nursing staff. In addition, a geriatrician and a clinical pharmacist provide a medication review for the patient. This geriatric consultation is also recorded in the patients’ digital medial file.

Delirium prevalence was not included as an outcome of this study, as the nursing staff may not record this diagnosis in a patient’s medical file; this may only be done by a physician or GNP. Physicians and GNPs diagnose delirium based on the DSM-IV criteria, often supported by information provided to them by the nursing staff. Management of delirium (for example reorientation strategies, mobilisation, hydration and sleep hygiene) are part of the nursing staff’s tasks to manage delirium. However, these management strategies are often not recorded in the patient’s medical file, and were therefore not included as an outcome of this study.

#### Demographic and background data

Demographic and background data from the nursing staff (gender, age, years of experience, and any previous delirium education) were obtained through a list of questions attached to the questionnaires. Demographic and background data for the patients (age, sex, length of stay (LoS, in days)), proportion of patients with a delirium, and duration of the delirium (DoD, in days)) were obtained through the patients’ digital medical files. LoS was counted as the number of days between the day of admission and the day of hospital discharge. DoD was counted from the date on which the diagnosis of delirium was first mentioned in the digital patient file, till, in order of importance: 1. a physician or a GNP noted in the patient files that the delirium was in remission or had passed; 2. pharmacological treatment for the delirium was ceased because of abating symptoms; 3. the DOS remained below three points for three consecutive measurements; 4. the patient had died during hospital admission; or 5. the patient had been discharged from the hospital.

#### Feedback

Lastly, we collected feedback from the nursing staff about the educational intervention during the second round of questionnaires on an extra page.

### Ethics and consent

The study was assessed and approved by the Medical Ethical Committee of the MUMC+ (#16–4-212). No consent was deemed necessary for the nursing staff, as the delirium training was considered as additional internal education the MUMC+ offers to its staff. No written consent for the patient data was needed, as in the Netherlands all university teaching hospitals have an opt-out system for the use of anonymous patient data for scientific research (i.e. patients automatically consent to the use of their anonymous data for research upon admission, unless they explicitly object).

### Analyses

Statistical analyses were carried out for two main outcome measures: proportion of patients screened using the DOS, and proportion of patients who received a geriatric consultation. No statistical tests were carried out for the proportion of patient with correct DOS scores due to the very low number of correct DOS scores pre-test (0–1). Demographic and background data of the patients were also not subjected to statistical testing, as these data were not expected to influence the main outcomes.

First data from the three pre-test time points (February 2016, May 2016 and February 2017) were compared to each other to check for any significant differences between these three time points to establish a baseline. If the pre-test data did not differ significantly between each other, we assumed that any significant changes found between the pre- and post-test situation could be attributed to the educational intervention. In this case, the data from all three pre-test moments were combined to form one pre-test group. In case significant differences would be found between the three pre-test time points, a stable baseline could not be assumed, and any differences between the pre-test and post-test conditions could not be automatically attributed to the intervention. The effect of the educational intervention on the proportion of patients screened using the DOS and proportion of patients who received a geriatric consultation were analysed using logistic regression. The basic model included time point (i.e. pre-test vs post-test), the hospital unit (i.e. A and B) and an interaction between time point and unit. If any of the variables or the interaction proved to be non-significant, the analysis was done again with only the significant variables in the model. Alpha was set at 0.05. Data was analysed using SPSS (IBM corp.) version 24.

## Results

### Participants

#### Nursing staff

A total of 34 nursing staff members participated in the- educational intervention: 16 (62%) staff members from unit A and 18 (55%) from unit B. Most of the participants were female (*N* = 31). The mean age was 34 (range 20–60), with an average work experience of 11 years (range 1–39). Five nursing staff members had had some delirium education in the last five years.

#### Patients

The files of 544 patients were included: 385 in the pre-test condition and 159 in the post-test condition. The three pre-test time points did not differ significantly from each other, thus the three pre-test time points were grouped together to form one pre-test group. See Table 1 for all demographic and background patient data.

**Table 1:** Demographic and background data of the patients

Time	Pre-test (*N* = 385)		Post-test (*N* = 159)	
Unit	A (*n* = 164)	B (*n* = 221)	A (*n* = 69)	B (*n* = 90)
Sex, %f	39.0	44.3	26.1	45.6
Age, m ± sd (range)	78.2 ± 5.9 (70–95)	79.5 ± 6.4 (70–99)	79.6 ± 5.6 (70–91)	80.3 ± 7.3 (70–101)
LoS, m ± sd (range)	6.9 ± 7.8 (1–64)	9.3 ± 10.2 (1–90)	7 ± 6.6 (1–37)	14.1 ± 12.8 (1–60)
Delirium, n(%)	19 (11.6)	31 (14.0)	8 (11.6)	13 (14.4)
DoD, n; m ± sd (range)	4.79 ± 2.3 (1–9)	8.5 ± 12.1 (1–68)	3.9 ± 2.9 (1–9)	16 ± 14.4 (1–49)

*LoS* Length of hospital stay (in days), *DoD* Duration of delirium (in days), *sd* standard deviation, *m* mean

### Main outcomes

The three outcomes were:

1. the proportion of older patients being screened using the DOS;
2. the number of DOS assessments being performed correctly; and
3. the proportion of patients being referred for a geriatric consultation.

A logistic regression was carried out to ascertain the effects of the educational intervention on the proportion of patients who were screened for delirium using the DOS. The variables “unit” and the interaction “unit x intervention” were deleted from the model after the first analysis because neither had a significant effect on the outcomes. Also, the main outcomes did not differ significantly between the time-points; therefore, the pre-test data was combined to form one pre-test group. Thus, the final regression model comprised only the intervention as independent variable.

**Fig. 2:**
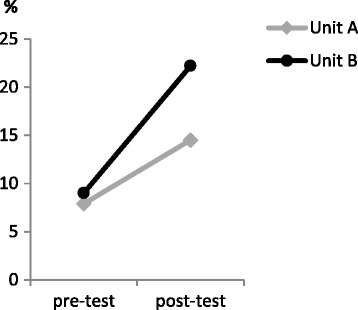
Proportion of older patients screened using the Delirium Observation Screening (DOS), separated per unit (A and B)

**Fig. 3:**
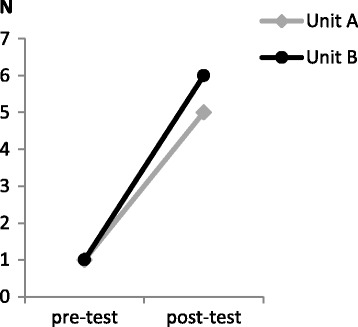
Number of DOS scores performed adequately (three times a day for at least three consecutive days) separated per unit (A and B)

The proportion of patients who were screened for delirium increased significantly in both units: from 7.9% to 14.5% in unit A and from 9.0% to 22.2% in unit B: for both units combined the odd’s ratio post-test versus pre-test = 2.481 (95% confidence interval = 1.454–4.231) *P* = .001 (see Table 2 and Fig. 2), indicating patients were about 2.5 times more likely to be screened for delirium after the educational intervention compared to before the educational intervention. Moreover, an increase in the number of patients for whom the DOS was performed correctly (i.e. three times a day for at least three consecutive days) on both units is noticed after the educational sessions, as shown in Table 2 and Fig. 3.

**Table 2:** Changes in clinical practice

Time	Pre-test (*N* = 385)		Post-test (*N* = 137)	
Unit	A (n = 164)	B (n = 221)	A (n = 69)	B (n = 90)
Using DOS^a^, n (%)	13 (7.9)	20 (9.0)	10 (14.5)	20 (22.2)
DOS correct ^b^, n	1	1	5	6
Geriatric consul-tation, n (%)	15 (9.1)	23 (10.4)	7 (10.1)	17 (18.9)

*DOS* Delirium Observation Screening scale

^a^Proportion of patients who were screened using the DOS

^b^Number of patients for whom the DOS was performed correctly (i.e. three times a day for at least three consecutive days)

**Fig. 4:**
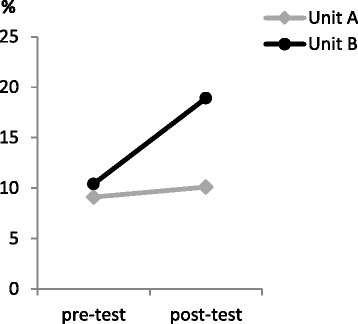
Proportion of older patients who received a geriatric consultation per unit (A and B)

A similar logistic regression model was used to analyse the effect of the intervention on the proportion of patients who received a geriatric consultation. This final model solely contained the variable of time (i.e. pre-test vs post-test). There was a slight though non-significant trend toward an increase in geriatric consultations after the educational intervention: odd’s ratio = 1.623 (95% confidence interval = 0.938–2.809) *P* = .083 (see Table 2 and Fig. 4).

### Feedback from the participants

Overall, the educational intervention was considered to be useful and insightful by the nursing staff; especially the use of videos, examples from clinical practice, and the lively discussion. Some participants missed practical guidelines on how to handle aggressive or very hyperactive patients.

## Discussion

The aim of this study was to assess the impact of an educational intervention for nursing staff on the clinical practice regarding screening and management of delirium in older hospitalised patients. We found a statistically significant increase in the proportion of patients screened for delirium using the Delirium Observation Screening score (DOS) in both studied units; patients were almost 2.5 times more likely to be screened for delirium with the DOS after the educational intervention had been delivered. Additionally, the number of patients for whom the DOS was performed correctly (i.e. three times a day for at least three consecutive days) increased notably in both units (from one correct DOS pre-test, to five or six post-test). The proportion of patients who received a geriatric consultation did not increase significantly after the educational intervention, though a trend towards more geriatric consultations was observed.

Although the number of patients who were screened correctly with the DOS increased after the educational intervention (from one per unit in the pre-test condition, to five or six per unit in the post-test condition), the clinical relevance of this finding may be questioned, as these numbers are small. However, it does indicate that a positive change occurred on both units with regards to the use of the DOS. It was beyond the scope of this study to assess whether the nursing staff adhered to the guidelines on how to complete the DOS, and therefore we have to be careful in interpreting these findings.

Moreover, it should be noted that there was no increase in the diagnosis of delirium on either ward after the educational intervention. Although this was not an outcome of the study, we believe it should be mentioned. The diagnosis of delirium is made by either a physician or a GNP, and may not be made by a nurse. Nursing staff can, however, inform the physician or GNP of a suspected delirium. Because nursing staff may not make the diagnosis, we did not include this as an outcome of our study (as the reporting of the diagnosis depends on a physician and not the nursing staff). We expect that the lack of increase in delirium prevalence is due to the fact that nursing staff is often told to keep DOS scores after a delirium has been diagnosed, instead of using the DOS as a screening instrument to detect early-stage or sub-syndromal delirium. However, we know from previous research that delirium often remains unrecognised by physicians, [2] and nurses also often miss a delirium when present. [3] Moreover, it is highly unlikely nurses will mistakenly identify a delirium when this is indeed not the case, [3] so it is probable that this study included patients who were delirious at the time of the study, but were not diagnosed as such (false negative).

It is difficult to compare our outcomes to previous studies, as very few studies on the education of nursing staff also examined changes in clinical practice. To our knowledge, just one study, a delirium e-learning programme for nurses conducted by Van de Steeg and colleagues, examined the proportion of older patients screened for delirium using the DOS [19]. This study included participants from 18 hospitals in the Netherlands, and also found a significant increase in the usage of the DOS and screening for frailty in older patients, which is consistent with our findings. Most other studies of educational interventions that have examined the implications for clinical practice were mostly interprofessional educational interventions for physicians and nursing staff [20–22], or were part of a multicomponent intervention [23, 24], and were not aimed only at the nursing staff. These studies showed that education combined with some reform of the regular care system (e.g. the introduction of a geriatric nurse or a care pathway) may increase delirium detection and improvement of delirium management. However, interprofessional education for physicians and nursing staff, including reorganisations or changes in care guidelines may be difficult to implement throughout a hospital.

Although the proportion and correctness of the screenings increased significantly after the educational intervention, we did not find a similarly positive result for the proportion of patients receiving a geriatric consultation. An important part of screening is the correct interpretation and evaluation of the DOS scores and consequently a correct course of action. In case of a delirium or suspected delirium, a geriatric consultation should be requested, or non-pharmacological interventions (such as a clock and calendar, mobilisation, cognitive stimulation and sleep hygiene protocols) could be used to manage the delirium [2]. Unfortunately, these interventions are often not accurately reported in a patient’s medical file, and could therefore not be included in this study as an outcome. Hence, it is difficult to assess whether and what the nursing staff exactly learned about delirium management from our educational intervention.

As can be observed from Fig. 3, the proportion of geriatric consultations increased more in unit B compared to Unit A. It might be the case that the sense of urgency for delirium was slightly less present for the nursing staff in unit A compared to unit B, or that the nursing staff in unit A was more reluctant to request a geriatric consultation for their patients, which may have led to the trend in increased geriatric consultations instead of a significant increase. Alternatively, it could be that although more patients were screened, this did not result in an increase of delirium diagnoses and consequently no increase in geriatric consultations.

Looking at the results, we noticed that the improvements in unit B were a bit higher than in unit A, though the results did not differ significantly from each other. These small differences could possibly be attributed to two things: the difference in patient population -unit A is a combination of many specialties, while unit B is a cardiology unit- and a difference in attitude or “culture” among the nursing staff in the two units. Anecdotally, during the educational sessions we perceived a greater willingness to cooperate from the staff on unit B, compared to the staff on unit A: more questions were asked, more patient situations and experiences were shared, and more feedback was provided during the educational sessions on unit B compared to unit A. Additionally, the leader of unit B did not make the delirium education mandatory, but the nursing staff was free to participate, while for unit A, the unit leader planned the delirium education as part of a mandatory staff meeting.

The willingness to cooperate and the openness for change could be considered very important aspects and possibly overlooked in educational interventions, including the present one. A change in behaviour is likely more prone to crystallise in an environment where this is encouraged and facilitated or driven by intrinsic motivation (staff members are free to attend the sessions), compared to externally enforced behaviour (a mandatory staff meeting). As such, it is important to take into account the attitude of the people for whom the education was designed, and, where possible, make sure the unit leaders understand the importance and added value of an educational intervention and openly support and encourage participation. Another option could be to appoint delirium “champions” or “key nurses”, who receive more rigorous delirium training, and can provide support to the rest of the nursing staff when dealing with delirious patients. Previous studies incorporating such champions have been found to bring about change in delirium detection and management [25], or can play a pivotal role in adapting new ways to work with older patients [26].

### Shortcomings and strong points of the study

We did not measure willingness to cooperate or attitudes towards delirium, which, in hindsight, might have given us more insight into why participants attended the educational sessions, and how well the learnt material is implemented in practice. Another important aspect to consider is the planning of the educational sessions. To facilitate participation of the nursing staff, we left it to each unit leader to plan the educational sessions. The staff members on unit B were free to attend the session if they wanted, though it was made clear that participation was greatly appreciated; the unit leader at unit A planned the educational session during a mandatory staff meeting, which had as a consequence that the educational session was also mandatory. These differences may have influenced the results of our study. Lastly, although we collected data from three different time points pre-test, we collected data for just one time point post-test. This was done for practical reasons, but it limits the conclusions we can make, as it might be possible that any differences we see after the intervention might not have consolidated on the long term, or the effect may have been the result of another external factor. We do not know, therefore, if the proportion and correctness of the screenings remained high several months after the educational intervention had taken place.

Our study also has several strong points. First, our primary outcomes were changes in clinical practice, instead of knowledge as assessed by the answers on a questionnaire. Rather, we used the questionnaires as a tool in our educational intervention: the content and focus of the educational session was based on the outcomes of the first assessment with the questionnaires and in the second round the questionnaires were used to reinforce the knowledge learned during the educational sessions. Another reason we decided not to include the questionnaires as a main outcome is that it cannot be guaranteed that the questionnaires were completed independently and without external help, rendering them unreliable as a study outcome. A second strong point in our methodology is the extended pre-test data collection: data was collected for three time points prior to the introduction of the educational intervention, so as to create a good baseline and avoid unjustly assigning any changes in clinical practice to the educational intervention when there might have been a pre-existing trend towards more screening or geriatric assessments. Moreover, because of the retrospective data collection, the nursing staff was not aware of the study during any of the pre-test time points, thus avoiding the possibility that they might have been triggered to perform better on any of the main outcomes.

## Conclusion

Overall, the educational intervention showed to have increased both the proportion of patients who were screened for delirium using the DOS as well as the number of patients for whom the DOS was used correctly. Moreover, a trend, though not significant, was observed for the proportion of patients who received a geriatric consultation from a GNP. The intervention was generally considered as positive and useful by the participating staff members. The educational sessions are easy to tailor, as there is a comprehensive base-presentation which can be adapted to meet the needs of the different hospital units. Although the intervention comprises several steps, it is feasible for hospital-wide implementation as there is only one component (the educational session) which requires the physical presence of the participants; the other components (questionnaires, posters, flyers) can be completed or consulted individually. Further research with a longer follow up time and larger sample is needed to establish if the results are just temporary or consolidate over time. Ideally, the educational programme should not only focus on the nursing staff, but also on physicians, as they have the final say about a patients’ treatment. Finally, unit leaders in hospitals should encourage the implementation of the learned material for optimal results.

## Abbreviations

DKQ: Delirium Knowledge Questionnaire
DoD: Duration of Delirium
DOS: Delirium Observation Screening score
GNP: Geriatric Nurse Practitioner
LoS: Length of Stay (in hospital)
M: Mean
MUMC+: Maastricht University Medical Centre+
PRECEDE model: Predisposing, Reinforcing and Enabling Constructs in Educational Diagnosis and Evaluation
SCDI: Strain in Care for Delirium Index
Sd: Standard deviation
